# The Man Who Pleases and the Woman Who Charms

**Published:** 1904-08

**Authors:** 


The Man Who Pleases and the Woman Who Charms. By John A. Cone. Sextodecimo, pp. 131. New York: Hinds & Noble. 1904. (Price, 75 cents.)

A book which gives the essence of good breeding in a nutshell is rather out of the ordinary for review in a medical journal; but there are physicians who would be better and more successful men and women by heeding the suggestions offered in this interesting little volume. Furthermore, it will not harm any person to read it, because it contains common sense teaching.

